# CovMulNet19, Integrating Proteins, Diseases, Drugs, and Symptoms: A Network Medicine Approach to COVID-19

**DOI:** 10.1089/nsm.2020.0011

**Published:** 2020-11-17

**Authors:** Nina Verstraete, Giuseppe Jurman, Giulia Bertagnolli, Arsham Ghavasieh, Vera Pancaldi, Manlio De Domenico

**Affiliations:** ^1^ Centre de Recherches en Cancérologie de Toulouse (CRCT), UMR1037 Inserm, ERL5294 CNRS, Toulouse, France.; ^2^ University Paul Sabatier III, Toulouse, France.; ^3^ MPBA Lab, Fondazione Bruno Kessler, Povo, Italy.; ^4^ CoMuNe Lab, Fondazione Bruno Kessler, Povo, Italy.; ^5^ Barcelona Supercomputing Center (BSC), Barcelona, Spain.

**Keywords:** COVID-19, disease network, symptoms, proteins, randomization, complex networks, interactome

## Abstract

**Introduction:** We introduce in this study CovMulNet19, a comprehensive COVID-19 network containing all available known interactions involving SARS-CoV-2 proteins, interacting-human proteins, diseases and symptoms that are related to these human proteins, and compounds that can potentially target them.

**Materials and Methods:** Extensive network analysis methods, based on a bootstrap approach, allow us to prioritize a list of diseases that display a high similarity to COVID-19 and a list of drugs that could potentially be beneficial to treat patients. As a key feature of CovMulNet19, the inclusion of symptoms allows a deeper characterization of the disease pathology, representing a useful proxy for COVID-19-related molecular processes.

**Results:** We recapitulate many of the known symptoms of the disease and we find the most similar diseases to COVID-19 reflect conditions that are risk factors in patients. In particular, the comparison between CovMulNet19 and randomized networks recovers many of the known associated comorbidities that are important risk factors for COVID-19 patients, through identified similarities with intestinal, hepatic, and neurological diseases as well as with respiratory conditions, in line with reported comorbidities.

**Conclusion:** CovMulNet19 can be suitably used for network medicine analysis, as a valuable tool for exploring drug repurposing while accounting for the intervening multidimensional factors, from molecular interactions to symptoms.

## Introduction

The recent years have seen the booming of the field of network medicine, a discipline that aims to exploit networks and their analysis to depict and understand the complex relationships between biological processes, drugs, phenotypes, and ultimately diseases.^1^

Never before has this approach been so relevant to the worldwide medical community, as doctors search for a cure for a novel disease, which appeared suddenly and quickly started making victims. COVID-19, the disease caused by infection with the SARS-CoV-2 virus, was officially named in January and since then the pace of science has been exceeding what we thought possible. Very fast patient data started being collected and hundreds of treatments were tried, some with more success than others, but none of them being able to prevent many deaths. Despite the debatable exact lethality of this disease, and the optimistic prospect of having a vaccine soon, the stress that treating these patients puts on health systems and the many unknowns regarding the exact pathology created by this virus contribute to make this by far the biggest medical challenge in recent times.

It is, therefore, interesting to see if all the tools that have been developed in network medicine for other diseases will help us better understand COVID-19 and also find better therapeutic options.

The most promising concept to find a treatment for a new disease is that of repurposing, that is, using a drug, or a combination of drugs, already approved for a different condition.^2^ This facilitates the approval of the treatment by the regulatory bodies as usage in humans is proven to be safe. The main general principle behind repurposing is that the same compound can be used for two diseases that are different but similar in some respect. Disease similarity has been described at many levels, either focusing on similarity of genetic alterations, of gene expression profiles, of symptoms and also of alterations of gene expression.^3^ All of these approaches lead to complex networks in which nodes can be proteins, drugs, diseases, or even patients. Commonly, diseases are represented as a network of interacting genes or proteins that are somehow altered in it.^4–6^

A possible approach to better understand COVID-19 is to assemble a COVID-19 network, starting from a basic understanding of the SARS-CoV-2 virus. This was possible thanks to pioneering work that experimentally mapped the interactions of the virus proteins with human host proteins.^7–9^ Knowing which human proteins can potentially interact with the virus allows us to describe a more complex network in which entire pathways and biological processes can be implicated in COVID-19 pathology.

A few articles have developed drug-repurposing strategies for COVID-19 starting from these initial works. Gordon et al. propose candidate drugs,^7^ Gysi et al. propose various ways of ranking drugs,^10^ and Sadegh et al. share an online tool to explore repurposing options interactively, as well as proposing a few examples of how to search for repurposing candidate drugs.^11^ Using expression from lungs of COVID-19 patients, Rian et al. identified specific pathways that are affected by SARS-CoV-2 infection and predicted the effect of 8000 compounds as potential treatments.^12^ An international effort is currently ongoing to organize and mine all available knowledge and data on this disease,^13^ its epidemiology,^14^ and to create accessible data repositories (https://github.com/CLAIRE-COVID-T4/covid-data).

Our understanding about the disease has greatly increased, and we now know that, contrary to initial reports, this pathology is far more than a respiratory disease, involving alterations of coagulation that can be just as deadly as the respiratory distress, which was one of the earliest identified causes of death associated to the virus.^15^

In this article, we construct CovMulNet19, a comprehensive COVID-19 network, obtained retrieving all available interactions involving SARS-CoV-2 proteins, their interacting-human proteins (from here on referred to as COVID-19 proteins), diseases and symptoms that are related to these human proteins, and compounds that can potentially target them. We then employ extensive network analysis methods based on a bootstrap approach to prioritize a list of diseases that display a specifically high similarity to COVID-19 and a list of drugs that could potentially be beneficial to treat patients affected by this disease.

Including symptoms in CovMulNet19 allows us to further characterize the pathology of the disease and to recapitulate many characteristic presentations such as respiratory failure, chest pain, nausea, and several neuronal dysfunctions.

We also found high similarity of COVID-19 to SARS as well as to pathologies of the intestine, liver, and neural system, in accordance with some of the identified risk factors. The integration of viral proteins, human proteins, diseases, symptoms, and drugs in an interactive visualization of this unified data set will enable the community to freely explore this disease in its molecular and medical context.

## Results and Discussion

### Constructing an integrated COVID-19 interactions network

**FIG. 1.**
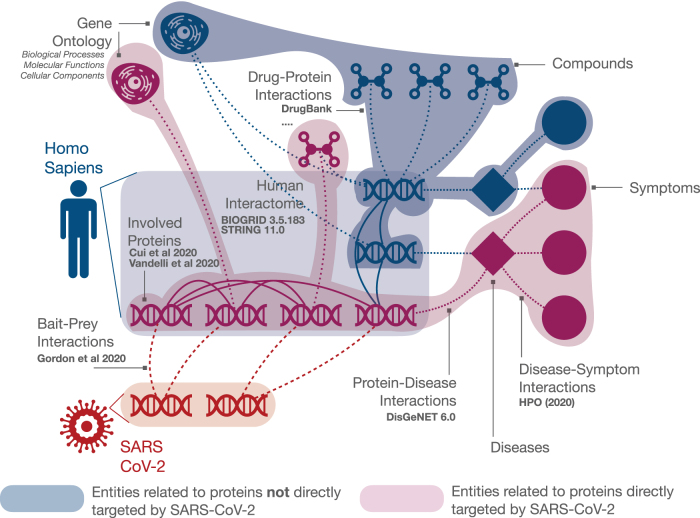
Linking genotype to phenotype in SARS-CoV-2–*Homo sapiens* molecular interactions. We build a highly reliable map of the human interactome and focus on the subset of human proteins that were shown to putatively interact with the virus in the literature, both through experimental protein interaction assays,^7^ through structure-based predictions,^9^ and based on similarity of the proteins to other coronaviruses proteins.^8^ The COVID-19 PPI network is enriched by biological information related to each involved protein (GO terms), as well as by an extensive data set of drug–protein interactions obtained by integrating different repositories. Finally, the system is enriched with phenotype information about diseases and symptoms, allowing us to include disease–symptom and protein–disease associations. Different icons represent different entities: genes, diseases, compounds, and symptoms are represented by DNA fragments, diamonds, chemical structures, and circles, respectively. Purple shaded area and purple icons represent entities associated with genes of human proteins directly targeted by SARS-CoV-2, whereas blue shaded area and blue icons denote entities related to genes of human proteins indirectly targeted by SARS-CoV-2 through human PPI. Cell icons represent GO terms, including biological processes, molecular functions, and cellular components. Solid lines highlight human PPIs and dotted lines represent other types of interactions between different entity types. See the text for details. GO, Gene Ontology; PPI, protein–protein interaction.

With the aim of summarizing available information on COVID-19 to enable network medicine analyses of this new pathology, we set out to collect information on interactions of the viral proteins with human proteins and the relationships between these proteins with diseases and symptoms. We expanded the set of experimentally validated SARS-CoV-2 interactors with predicted interactions (see Materials and Methods section) and proceeded to reconstruct the human Protein–Protein Interaction (PPI) network that is potentially affected by the virus. To this end we combined functional interactions from STRING database^16^ with experimentally detected physical and genetic PPIs from BioGRID.^17^ We then explored how these proteins are related to specific diseases as annotated in the DISGENET database, which lists genes associated with diseases mainly through mutations. We then integrated data from six different drug–protein interaction databases into our network, to provide a set of close to 6000 compounds that could be potential repurposing candidates. Finally, and most importantly, we added interactions between proteins and symptoms, using the Human Phenotype Ontology (HPO^18^), which allows us to identify specific connections between SARS-CoV-2 proteins, human proteins and the different manifestations of COVID-19. To facilitate the user in the exploration of the resulting integrated network, we have added Gene Ontology (GO) terms corresponding to each human protein as nodes in the network. Figure 1 shows an overview of the network construction procedure.

**FIG. 2.**
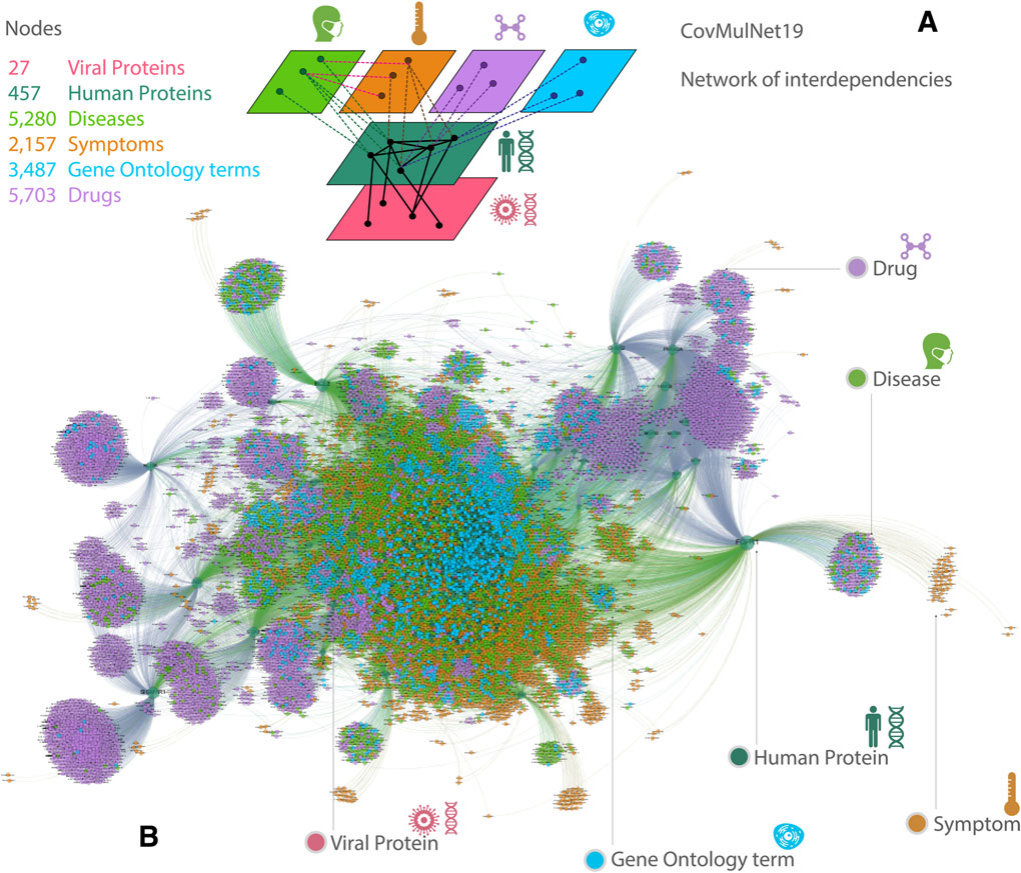
CovMulNet19 COVID-19 genotype–phenotype–drug interaction network. Result of the data integration and processing procedures illustrated schematically in Figure 1. **(A)** Nodes and schematic map of interdependencies among different layers encoding diseases, symptoms, drugs, GO terms, human proteins, and viral proteins. **(B)** Map of the reconstructed structural interactions (e.g., protein–protein) and functional interdependencies (e.g., protein–disease, protein–GO term, or disease–symptom). Overall, the network consists of 1999 protein–protein, 19,755 protein–disease, 10,152 protein–symptom, 13,018 drug–target, 9210 protein–GO, and 3056 disease–symptom relationships.

The final result of this network construction comprises 27 viral genes, 457 human proteins, 5280 diseases, 2157 symptoms, 3487 GO terms, and 5703 drugs. It is composed of 17 connected components, among which the largest connected component is made of 19,892 nodes, including the 457 viral protein interactors and representing 99.81% of the network. Figure 2 shows a visual representation of our multidimensional network that can also be interactively explored at https://covmulnet19.fbk.eu/.

### Identifying unique features of CovMulNet19

To test whether this network captures some specific aspects of COVID-19, we investigated whether the set of human proteins that interact with SARS-CoV-2 proteins have specific functional roles, are associated to specific diseases and symptoms or can be targeted by specific drugs, differently from equally large sets of randomly chosen human proteins. We hypothesize that finding the unique connections of COVID-19 to diseases, drugs, and symptoms will help identify valid repurposing options for its treatment that will specifically target this pathology. Moreover, this prevents us from overestimating the importance of diseases or symptoms that simply interact with many human proteins and appear in our CovMulNet19 only for this reason, validating the specificity of our findings for COVID-19.

We performed a degree analysis on CovMulNet19 to identify diseases and symptoms that interact with many of the COVID-19 proteins, and potential drugs that could represent valuable candidate COVID-19 treatments. This approach builds on the principle that if a drug can target multiple SARS-CoV-2 viral protein interactors specifically, it might hit many of the mechanisms the virus uses to attack the host.

To identify which disease, symptoms, and drug nodes of the network are particularly important in COVID-19 pathology, we used a bootstrap resampling method to evaluate whether the nodes with a high degree in CovMulNet19 were not simply highly connected because they represent hubs in all known protein networks from public interactomes, which would lead these nodes to be also highly connected in any random network. In contrast, we considered that the nodes with a higher degree in CovMulNet19 than in random networks were potentially medically relevant. We generated 2500 mock networks composed of 457 random proteins from the BIOSTR database applying the same method as we used in the creation of CovMulNet19 to find associations with GO terms, diseases, symptoms, and drugs for these sets of random proteins. The mock networks contain between 212 and 654 (average 384.5) PPIs, compared with 1999 PPIs in CovMulNet19. This is evidence of the coherence of proteins that interact with the virus, including multiple members of the same specific pathway or protein complexes.

**FIG. 3.**
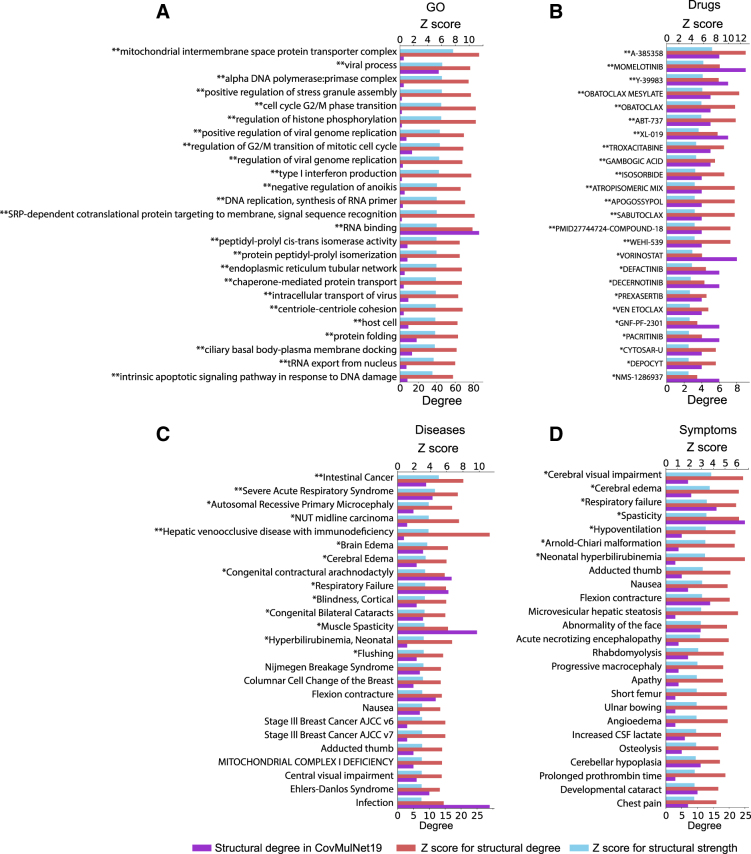
Top 25 over-represented GO terms, drugs, diseases, and symptoms in CovMulNet19. The 25 most over-represented GO terms **(A)**, drugs **(B)**, diseases **(C)**, and symptoms **(D)** are ranked based on their z-scores calculated on structural strength using the bootstrap sampling procedure. The top *X*-axis shows z-score values and bottom *X*-axis shows structural degrees (nodes degrees to protein nodes). Red and blue bars depict z-scores calculated on the structural degrees and on their structural strength (i.e., degrees to proteins relatively to the total degrees to proteins from all nodes), respectively. Purple bars represent the nodes' structural degrees observed in CovMulNet19's network. Terms preceded with a (*) or (**) were significantly over-represented in CovMulNet19 compared with observed appearance in the mock random networks (*p*-value <0.1 and 0.05, respectively). The complete list of nodes with their associated z-scores and p-values can be accessed in the bootstrap results tables in supplementary data (Supplementary Tables S1 and S2).

Degrees were calculated for all nodes as the number of edges to human proteins (either putative SARS-CoV-2 interactors in CovMulNet19 or random proteins in the mock networks). We define the *structural degree* as the number of connections of each node to human proteins and the *structural strength* as the ratio of the structural degree to the total number of connections to proteins in the considered network (in a node-type dependent manner). Z-scores were then calculated and used to evaluate the over- and under-representation for each node in CovMulNet19 compared with what was expected at random based on results on the mock data sets (Fig. 3).

### CovMulNet19 highlights potentially medically relevant aspects of COVID-19

Figure 3 shows over-representation of GO terms, drugs, diseases, and symptoms after bootstrap bias correction and degree analysis. The GO terms that are over-represented in CovMulNet19 compared with the mock networks highlight biological processes, molecular functions, and cellular components consistent with the possible roles of SARS-CoV-2 interacting human proteins in the viral infection process. These include viral processes (PABPC1 role in the positive regulation of coronavirus genome replication^19^), immune processes (roles of TBK1 and IRF3 in Type I interferon production^20^), RNA and DNA metabolism (RAE1 role in tRNA export from nucleus,^21^ DNA replication stress induced by coronavirus infection^22^) and mitochondrial transport (Translocase inner mitochondrial membrane subunits and their role in antiviral immunity^23^).

The diseases that are over-represented in CovMulNet19 compared with the mock networks based on their z-scores, include SARS and other respiratory, intestinal, liver, and neurological diseases or conditions, as well as a blood cancer, consistent with COVID-19 pathology and risk factors highlighted by recent meta-analyses.^24^ Interestingly, symptoms that are over-represented in CovMulNet19 compared to the mock networks according to their z-scores, also include respiratory failure, nausea, and other neurological conditions. From the start, the list of COVID-19 symptoms included respiratory issues and nausea, but there are increasing reports of neurological symptoms that had been overlooked in the first few weeks of the epidemic that can be typical of other virus infections or quite specific.^25^ Finally, among the drugs targeting a high number of SARS-CoV-2 interacting human proteins, we found many BCL-2 inhibitors (A-385358, Obatoclax Mesylate, ABT-737, Apogossypol, Sabutoclax), which suggests that inhibiting this protein, thus controlling the related antiapoptotic pathways, might be beneficial to COVID-19 patients. Interestingly, BCL-2 is targeted by some of the treatments for leukemias, which incidentally share some of the less specific symptoms of COVID-19 such as fatigue, fever, and nausea. Several studies proposed that BCL-2 inhibitors could also be repurposed for antiviral drug development.^26,27^ Although the mechanisms at play remain to be unveiled, the authors of these studies suggested that infected cells might release proapoptotic proteins from BCL-xL to initiate mitochondrial membrane permeabilization, adenosine triphosphate degradation, and caspase-3 activation. Subsequent treatments with BCL-2 inhibitors drove apoptosis of the infected cells. However, these treatments might need to be evaluated individually, as they might need to be combined with other drugs modulating the inflammatory response or promoting viral clearance as another study reported altered proinflammatory cytokine profile in the lung and a slightly higher viral load in influenza virus-infected mice treated with ABT-263.^28^ In addition, several Janus kinase inhibitors have also been included in clinical trials to treat COVID-19 patients admitted to hospitals,^29–31^ and we find two drugs from this category among our top hits (Momelotinib, XL-019).

Taken together, these observations point to the potential of our approach to highlight relevant drug-repurposing candidates and also to explain some of the most mysterious symptoms of COVID-19 by highlighting this disease's similarities with other pathologies. The strong connection between COVID-19 and the immune system might be at the origin of the similarities between this new pathology and tumors of the blood and the state of overall body-wide inflammation observed in patients.

### Limitations of the current approach

Despite our best efforts in collecting all available information at the time of writing, this virus and associated pathology remain new and mostly uncharacterized. The availability of an interactome involving human and viral proteins has been a game changer, but it is clear that even experimental interaction assays have biases and a high level of false positives and negatives. To begin with, the interactions were assessed inside a human cell line with plasmid-based expression of the bait proteins, meaning that the physiological relevance of the observed interactions is not guaranteed inside any cell of the human body. The addition of predicted interactions clearly increases the chances that some of the edges included in the network might not be real. For this reason, we have repeated the entire analysis using exclusively the 332 proteins that were experimentally detected by Gordon et al.^7^ and we have included the corresponding results in Supplementary Tables S1–S4. As can be seen in the Supplementary Tables S3 and S4, most of the results remain unchanged, indicating that the further inclusion of the 125 proteins from predicted interactions does not substantially alter our findings, and might even increase their specificity toward SARS-CoV-2 pathology, since, for example, “Severe Acute Respiratory Syndrome” appears to be the second most over-represented disease only after adding these predicted interactions and is only found at position 1198 of the ranking with a negative z-score of −0.14682 in the analysis using exclusively experimental interactions. Moreover, we must also consider that inaccuracies generally plague large-scale databases of proteins/drugs/diseases interactions, both due to the data being inaccurate and to issues in the merging of different identifiers and simple human errors. Overall, the bootstrap approach presented in this study and the recapitulation of most of our results with a data set considering only experimentally validated interactions, should ensure that our findings are robust and do not rely on just a few specific network edges (which could represent false positives in the network's interactions). CovMulNet19 should only be viewed as a tool for hypothesis generation and any suggestion for biologically relevant associations between COVID-19 and genes, drugs, diseases, or symptoms should be experimentally verified before being considered further.

## Conclusion

Overall, the analysis presented in this study shows that CovMulNet19 can be suitably used for network medicine analysis, as a valuable tool for exploring drug repurposing while accounting for the intervening multidimensional factors, from molecular interactions to symptoms. The result of the comparison between CovMulNet19 and randomized networks recovers many of the known associated comorbidities that are important risk factors for COVID-19 patients, through identified similarities with intestinal, hepatic, and neurological diseases as well as with respiratory conditions, which is in line with reported comorbidities.^24^ Interestingly, focusing on the different components of CovMulNet19, we can explore the mechanistic connection between SARS-CoV-2 proteins, human proteins, other diseases, and symptoms, with a view toward more specifically targeting biological processes altered by COVID-19.

## Materials and Methods

### Building the human interactome: BIOSTR

In this section, we provide details about the procedure used to reconstruct the interaction network of human proteins by cross-linking different publicly available databases.

Since databases do not use the same format for protein names, as a first step we used the NCBI gene database to map all protein names and aliases to a common nomenclature of official symbols. Specifically, we used the data made publicly available from NCBI at the URL ftp://ftp.ncbi.nlm.nih.gov/gene/DATA/GENE_INFO/Mammalia/(Accessed March 28, 2020).^32^

In a second step, we downloaded two PPI networks for *Homo sapiens*. More precisely, we considered BioGRID v3.5.182^17,33^ (publicly available at URL: https://downloads.thebiogrid.org/BioGRID/Release-Archive/BIOGRID-3.5.182/) and the STRING v11.0^16^ functional interactions network (publicly available at URL: https://string-db.org/cgi/download.pl).

In the BioGRID data, we filtered by official (common) symbols for proteins, identifying a total of 429,232 PPIs. A total of 30,959 interactions (7.21% of the data set) contained at least one protein with noncommon symbol. After discarding the later interactions, a total of 18,053 proteins (nodes) and 398,273 interactions (edges) were identified. The resulting BioGRID network of interactions exhibits a multilayer structure,^34,35^ including different biologically relevant layers^36–38^: (1) direct interaction, (2) physical association, (3) suppressive genetic interaction defined by inequality, (4) association, (5) colocalization, (6) additive genetic interaction defined by inequality, and (7) synthetic genetic interaction defined by inequality. For the following analysis, we will consider the aggregated representation of this multilayer functional PPIs.

In the STRING data, we filtered high-confidence interactions with any type of evidence (score >0.7), identifying a total of 17,161 proteins and 839,522 PPIs out of the original data—including low-confidence interactions—consisting of 11,759,454 PPIs among 19,566 proteins. No biological layer classification is performed on this data set.

The merging of the two distinct networks was performed by applying the union of the corresponding sets of PPIs and the final result is named BIOSTR. Overall, the merged interactome—after removing duplicated PPIs—consists of 19,945 proteins and 737,668 high-confidence and undirected PPIs. Therefore, BIOSTR is more complete than BIOGRID and STRING separately, complementing them with 10.5% and 16.2% more proteins, respectively. Note that, *a posteriori*, filtering the BIOSTR network data by the NCBI map described earlier results in about 900 less proteins, since some names are not recognized as official.

### Building the human genotype–phenotype interactome

We gathered information about gene–disease interactions from DISGENET v6.0^39^ (publicly available database at the URL: https://www.disgenet.org/downloads) and filtered genes by the ones in our BIOSTR interactome, thus excluding associations involving proteins not in our PPI network. All types of sources were included: curated (UniProt, PsyGeNET, Orphanet, the Cancer Genome Interpreter, Comparative Toxicogenomics Database (CTD) (human data), ClinGen, and the Genomics England PanelApp), from animal models (Rat Genome Database, Mouse Genome Database, and CTD [mouse and rat data]) and inferred (HPO, and GDAs inferred from Variant-Disease Associations reported by Clinvar, the GWAS catalog and GWAS database). We considered all gene–disease associations with no further filtering based on scores. See https://www.disgenet.org/dbinfo#score for more details.^39^ Each disease found in the filtered DISGENET database was associated to symptoms found in the HPO (accessed on March 2020)^18^ publicly available at the URL: https://hpo.jax.org/app/.

Note that even if DISGENET provides a mapping to other databases, including the HPO and the Disease Ontology (DO), cross-linking with the DO data is very restrictive and we opted for the HPO. The main issue of this choice is to link DISGENET diseases identifiers to symptoms in the HPO: we used Unified Medical Language System identifiers available in DISGENET to link cross-references in the HPO. The final network consists of 15,228 HPO symptoms (nodes) and 628,686 gene–disease associations (edges) in DISGENET among which we found 598,556 matching symbols in our BIOSTR. Among the 96,745 diseases in DISGENET, a subset of 5280 was identified as being related to COVID-19 given their interaction with COVID-19 proteins, together with a set of 2157 symptoms. For each gene–disease–symptom interaction identified, a link between the gene and the symptom was added.

### Enhancing proteins metadata with GO information

For each protein in our BIOSTR PPI network, we searched for functional information by connecting it to terms in the GO publicly available at the URL: http://geneontology.org/docs/download-ontology/(go.obo and goa_human.gaf data sets).^40,41^ This information is added to the multidimensional system in terms of gene-biological class relationships, including all GO terms (molecular function, biological process, and cellular component). In total, proteins from BIOSTR concern 30,657 biological processes, 12,134 molecular functions and 4431 cellular components.

### Building the SARS-CoV-2 virus–host interactions

We started from the molecular interactions of SARS-CoV-2 with human proteins (virus–host interactions) identified by affinity-purification mass spectrometry by Gordon et al.^7^ The identified bait–prey interactions consist of 22,153 unthresholded links, with 332 (1.5%) above the threshold suggested by Gordon et al. We have further expanded this subset of the human proteome involved with COVID-19 by including 113 proteins predicted to be related by Vandelli et al. through homology^9^ and 30 proteins found by Cui et al. from analyses across >2500 coronaviruses.^8^ The overall number of proteins considered in our virus–host interaction network is 457, after filtering for duplicated protein aliases.

### Building the drug–target interactions

The interactions between a chemical compound (or a drug) and its protein targets were collected from six publicly available data sources. The definition of interaction is heterogeneous across different sources, and thus, for each database, we explicitly list hereafter the corresponding definition. Note that some drug nodes are reported in terms of their combination with other drugs, for example, “G3139 + DEXAMETHASONE.”

DrugBank v.5.1.5^42^ (https://www.drugbank.ca/): A target is defined as a protein, macromolecule, small molecule, and so on to which a given drug binds or otherwise interacts with, resulting in an alteration of the normal function of the bound molecule and desirable therapeutic effects or unwanted adverse effects.

DGIdb v.3.0.2^43^ (http://www.dgidb.org/): Here a drug–gene interaction is defined by the database curators as a known interaction (e.g., inhibition) between a known drug compound (e.g., lapatinib) and a target gene (e.g., EGFR).

Therapeutic Target Database v.11 November 2019^44^ (http://db.idrblab.net/ttd/): Interactions are defined as connections between known and explored therapeutic protein targets and the corresponding drugs directed at each of these targets.* Note that some drugs in the data set are reported in terms of their combination with other drugs.

Drug Target Commons^45^ (http://drugtargetcommons.fimm.fi/): Interactions are defined as annotated or unannotated bioactivity between drug and target.

chEMBL v.26^46^ (https://www.ebi.ac.uk/chembl/): Interactions are known pharmaceutical associations as declared by drug producers. chEMBL also provides annotated experimental drug–target interactions that were not included in CovMulNet19.^†^

Tabei et al.^47^ (http://labo.bio.kyutech.ac.jp/~yamani/drugprotein/): The links are a subset of 78,692 drug–protein interactions extracted from older versions of ChEMBL,^48^ KEGG,^49^ DrugBank,^50^ PDSP Ki,^51^ and Matador.^52^

The original sources adopt the following nomenclature for the drug ID (as reported from the corresponding official information):

- DrugBank—*Standard name of drug as provided by drug manufacturer*
- Tabei DB—*Drugbank ID*
- DGIdb—*the primary drug name*
- Therapeutic Target Database—*Drug Name*
- Drug Target Commons—*Compound name*
- ChEMBL—*Compound name and synonyms.*

The harmonization of the drug identifier was thus needed, by mapping on the BioGrid reference.

### Integrating the genotype–phenotype network with drugs

We cross-linked the gene–disease interactions with the drug–target interactions described in the previous sections to obtain an overall map linking molecular interactions to phenotypes related to COVID-19 in *Homo sapiens*. Finally, the overall network consists of 27 viral genes, 457 human proteins, 5280 diseases, 2157 symptoms, 3487 GO terms, and 5703 drugs. See Figure 2 for a visual representation of our multidimensional network, which can also be interactively explored at https://covmulnet19.fbk.eu/.

### Bootstrap analysis

A total of 2500 sets of 457 proteins chosen randomly from those included in the BIOSTR database were used to create mock data sets comparable with CovMulNet19. Degrees were calculated for all nodes as the number of edges to human proteins (either putative SARS-CoV-2 interactors in CovMulNet19 or random proteins in the mock networks). We define the *structural degree* as the number of connections of each node to human proteins and the *structural strength* as the ratio of the structural degree to the total number of connections to proteins in the considered network (node-type dependent). Z-scores were calculated according to the standard formula $Z=\frac{x-\mu }{\sigma }$, with *x* being the structural strength (or structural degree) of a node measured in CovMulNet19, and *μ* and *σ* being the mean structural strength (or mean structural degree) and the standard deviation structural strength (or standard deviation structural degree) of the same node across the random networks where it was found, respectively. *p*-Values were then calculated for each node based on the obtained z-scores and the normality of the structural degrees or structural strengths distributions across mock networks. When normally distributed, *p*-values were calculated with $p=1-erf\left(\frac{\left|Z\right|}{\sqrt{2}}\right)$, and adjusted to 0.5 for null z-scores. When not normally distributed, we used Chebyshev's inequality with $p=\frac{1}{Z^{2}}$, and adjusted *p*-values to 1 for |*Z*| ≤ 1. Finally, the calculated z-scores and corresponding *p*-values were used to evaluate the over- and under-representation for each node in CovMulNet19 compared with what was expected at random based on results in the mock data sets, allowing us to identify the top ranking gene ontology terms, diseases, drugs, and symptoms in CovMulNet19 compared with the mock random data sets (Fig. 3 and Supplementary Tables S1 and S2).

## Data Availability

The CovMulNet19 data set consists of two text files, named COVID19-GDDS457-nodes and COVID19-GDDS457-edges, respectively, both in csv format, deposited on the public repository FigShare and publicly available at the web addresses https://figshare.com/articles/CovMulNet19_zip/12563192/2.

The first file includes the 17,111 biological entities representing the nodes of the CovMulNet19 network. Each row has three columns, detailing the node name, an integer code for the node type, and the node type description, with the following notation:

**0** Viral Gene;

**1** Human PPI (target);

**3** Disease;

**4** Symptom;

**5** Drug;

**6** GO.

The second file includes the 57,526 interactions between pairs of nodes: each row consists of three comma-separated columns, with the names of the two nodes being linked and their type of association (disease–symptom, human PPI (target)–drug, human PPI (target)–GO, etc.).

We decided to share CovMulNet19 in a flat text file format to maximize its usability within different analytical frameworks and to allow its easy visualization on multiple platforms.

Apart from the data set, we provide access to an interactive dashboard at the https://covmulnet19.fbk.eu/allowing to visually explore the CovMulNet19 network and its metadata.

## Code Availability

The data set was generated by open source frameworks (R and Python) processing publicly available data sets. The source code creating the network is available upon request to the corresponding author.

## Supplementary Material

Supplemental data

Supplemental data

Supplemental data

Supplemental data

## Abbreviations Used

CTD: Comparative Toxicogenomics Database
DO: Disease Ontology
GO: Gene Ontology
HPO: Human Phenotype Ontology
PPI: protein–protein interaction

## Notes

^*^ TTD was updated on June, the 1st 2020 while drafting the current manuscript. New interactions have not been added to **CovMulNet19**.

^†^ chEMBL was updated on May, the 21st 2020 while drafting the current manuscript. New interactions have not been added to **CovMulNet19**.
